# Thoracoscopic lobectomy for lung cancer in a patient with a partial anomalous pulmonary venous connection: a case report

**DOI:** 10.1186/s13019-016-0527-7

**Published:** 2016-08-02

**Authors:** Kenji Inafuku, Takao Morohoshi, Hiroyuki Adachi, Keisuke Koumori, Munetaka Masuda

**Affiliations:** 1 Department of General Thoracic Surgery, Yokosuka Kyosai Hospital, 1-16 Yonegahamadori, Yokosuka, Kanagawa 238-8558 Japan; 2 Department of Surgery, Yokohama City University Graduate School of Medicine, 3-9 Fukuura, Kanazawa-ku, Yokohama, Kanagawa 236-0004 Japan

**Keywords:** Lung cancer, Partial anomalous pulmonary venous connection, Video-assisted thoracoscopic surgery, Case report

## Abstract

**Background:**

A partial anomalous pulmonary venous connection is a rare congenital defect in which blood from the pulmonary vein is returned to the right atrium. Asymptomatic patients with a partial anomalous pulmonary venous connection with a small left-to-right shunt do not require surgical treatment. If such patients require a major lung resection, the surgical procedure could precipitate fetal right heart failure if the anomalous venous connection remains uncorrected.

**Case presentation:**

A 59-year-old man was found to have an abnormal shadow on chest roentgenogram. Chest computed tomography imaging showed a mass in the right upper lobe. At the same time, we incidentally found an anomalous vessel. We diagnosed the abnormality as a partial anomalous pulmonary venous connection. Because the mass may have been lung cancer, a right upper lobectomy was performed using video-assisted thoracoscopic surgery. The right upper lobe vein drained into the superior vena cava. The anomaly was not corrected and the surgery was successful. His postoperative course was uneventful without cardiac failure.

**Conclusions:**

Before performing a major lung resection, surgeons should be aware of this rare anomaly and carefully interpret clinical images of all pulmonary veins.

## Background

A partial anomalous pulmonary venous connection (PAPVC) is a rare congenital defect in which the right atrium is the point of return for blood from the pulmonary vein. This anomaly is often associated with other congenital heart defects, especially atrial septal defects. Asymptomatic patients with a PAPVC with a small left-to-right shunt do not require surgical treatment. However, if patients with PAPVC require a major lung resection, the surgical procedure could precipitate fetal right heart failure if the PAPVC is not corrected [1, 2]. In this article, we present a case of right PAPVC that was diagnosed during preoperative examinations for lung cancer.

## Case presentation

**Fig. 1:**
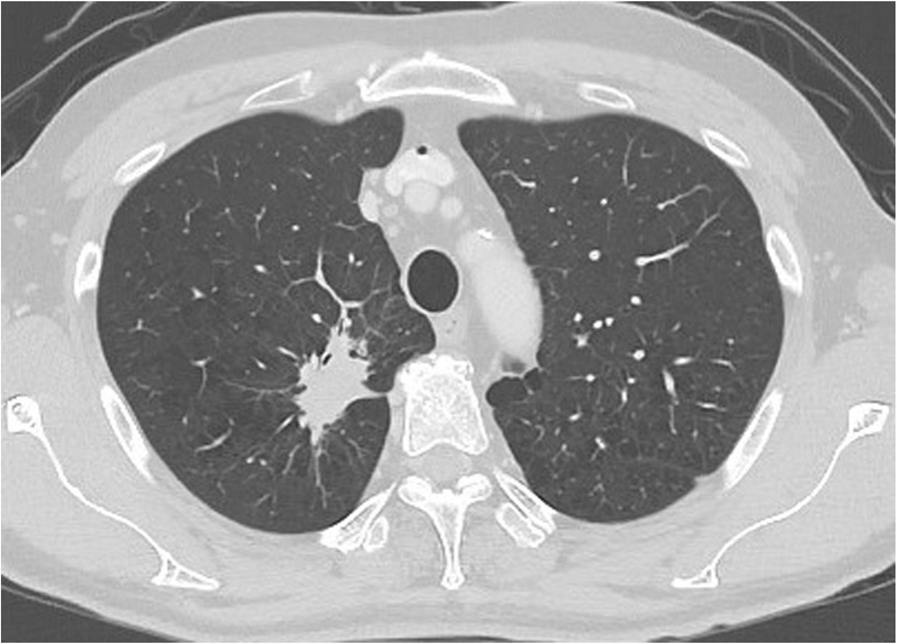
Computed tomography image. The chest computed tomography image shows an irregular mass in the S1 segment of the right lung

**Fig. 2:**
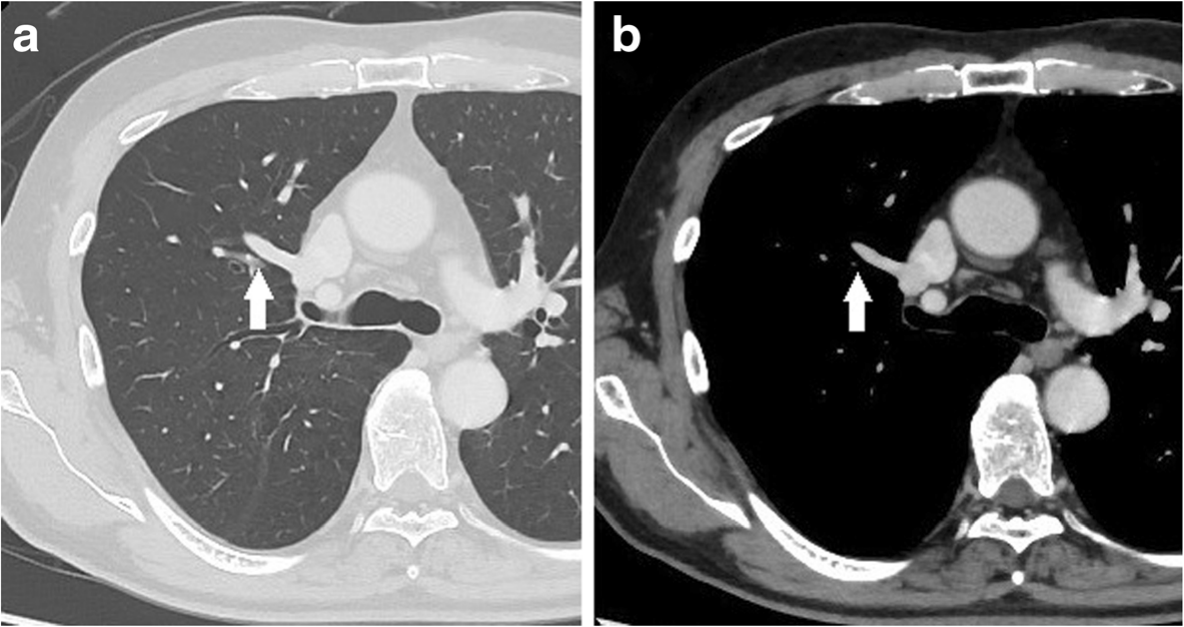
Computed tomography image. The chest computed tomography image shows an enhanced anomalous vein that continues from the superior vena cava (*arrow*). **a** The mediastinal window. **b** The lung window

An abnormal shadow was detected in a 59-year-old man during a routine medical examination. He was asymptomatic and his past history was unremarkable. The chest roentgenogram showed a mass shadow in the right upper lung field. Chest computed tomography (CT) imaging showed a 4.5 cm × 4.0 cm mass in the S1 segment of the right lung (Fig. 1). At the same time, we incidentally found an anomalous vessel. This vessel originated from the right upper lobe and drained into the superior vena cava (SVC) (Fig. 2). We diagnosed the abnormality as a PAPVC. Positron emission tomography–computed tomography using ^18^F-fluorodeoxyglucose (FDG) showed a nodule with maximum standard uptake value of 10.6. The echocardiogram showed normal cardiovascular activity without an atrial septal defect. The preoperative blood gas analysis revealed a partial pressure of arterial oxygen (PaO_2_) of 77.1 mmHg and partial pressure of arterial carbon dioxide (PaCO2) of 41.3 mmHg in room air. Spirometry revealed normal respiratory function.

**Fig. 3:**
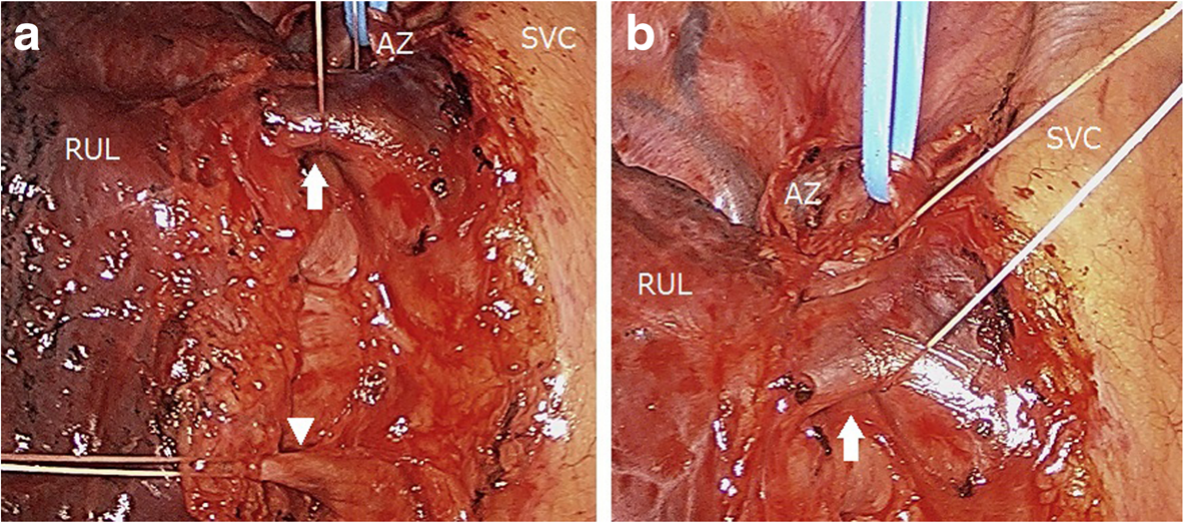
Intraoperative thoracoscopic view. Intraoperative findings. The thoracoscopic view shows the anomalous right upper lobe vein as it continues from the superior vena cava (*arrow*). The pulmonary vein (V2t) is normally connected to the left atrium (*arrowhead*). **a** The distant view. **b** The near view. AZ, azygos vein; RUL, right upper lobe; SVC, superior vena cava

Because the mass may have been lung cancer—and if so, it was believed to be a resectable lesion with a clinical staging of T2aN0M0 stage IB—a right upper lobectomy was performed using video-assisted thoracoscopic surgery. The anomalous pulmonary vein drained into the SVC. The pulmonary vein (V2t) was normally connected to the left atrium (Fig. 3). Based on needle biopsy, the lesion was diagnosed as non-small cell lung cancer. A right upper lobectomy and dissection of the lymph-node were curative. The anomalous vein was mechanically cut and sutured using a stapler. We thereafter ligated and cut the normal V2t. The surgery was completed successfully. His postoperative course was uneventful without any cardiac failure. The postoperative blood gas analysis revealed a PaO_2_ of 72.1 mmHg and a PaCO_2_ of 39.1 mmHg in room air. In the resected specimen, the tumor measured 4.7 cm. It was histopathologically an invasive adenocarcinoma. The pathologic stage of the lung cancer was IB (i.e., T2aN0M0). Four months after the surgery, he remains in good health without relapse of lung cancer.

## Discussion

A PAPVC is a relatively uncommon congenital anomaly that is detected in only 0.4–0.7 % of the general population at the time of autopsy [1–3]. These anomalies are often associated with other congenital heart defects, especially atrial septal defects, in 80–90 % of patients [4]. A PAPVC results in the recirculation of oxygenated pulmonary venous blood through the pulmonary veins (i.e., left-to-right shunt).

Surgical treatment of a PAPVC is recommended for patients with a Qp/Qs greater than 2.0, regardless of associated cardiac defects [5]. In practice, the amount of left- to-right shunt is usually small; therefore, clinical manifestations such as fatigue, dyspnea, syncope, atrial arrhythmias, right heart failure, and pulmonary hypertension rarely occur [4]. However, in patients with lung cancer, this anomaly may present some serious problems. If lung cancer is in a lobe that has the abnormal vein, surgical resection is sufficient treatment. On the other hand, if the PAPVC is in a different lobe, a major lung resection could increase the volume of shunt flow and cause right-sided heart failure. Therefore, it is critical to correct this anomaly to prevent heart failure.

In patients with lung cancer, a PAPVC on the left side is corrected by a simple end-to-side anastomosis to the left auricular appendage or the left atrium, or by an end-to-end anastomosis to the stump of the resected normal pulmonary vein without extracorporeal circulation [6]. When the PAPVC is located on the right side, a cardiopulmonary bypass is usually required because of the shortness of the anomalous vein.

Black and associates [1] reported a patient who had fetal right heart failure after undergoing right pneumonectomy for lung cancer with a missed contralateral PAPVC. Sakurai and associates [7] reported a case of right PAPVC repair using cardiopulmonary bypass before left pneumonectomy; the patient had an uneventful course. Takei and associates [8] similarly reported a case of an anomalous pulmonary vein of the left upper lobe that was separated from the left brachiocephalic vein and anastomosed to the stump of the inferior pulmonary vein.

Therefore, before performing a major lung resection, surgeons must always consider the possible presence of a PAPVC and carefully interpret the CT imaging of all pulmonary veins. If an anomalous vein is incidentally detected during a surgery for lung cancer or the patient develops unexplained fetal right heart failure after the surgery, it is important to reinterpret the preoperative CT images and discuss carefully whether to perform a revascularization procedure while considering a PAPVC.

## Conclusions

We experienced a patient with a PAPVC and primary lung cancer in the same lobe, which was successfully treated by lobectomy. Before performing any major lung resection, surgeons should be aware of this rare anomaly and carefully interpret the clinical images of all pulmonary veins of the resected and the nonresected pulmonary lobes.

## Abbreviations

CT, computed tomography; FDG, ^18^F-fluorodeoxyglucose; PaCO2, partial pressure of arterial carbon dioxide; PaO_2_, partial pressure of arterial oxygen; PAPVC, partial anomalous pulmonary venous connection; SVC, superior vena cava
